# Prognostic Roles of mRNA Expression of S100 in Non-Small-Cell Lung Cancer

**DOI:** 10.1155/2018/9815806

**Published:** 2018-01-21

**Authors:** Ying Liu, Jian Cui, Yun-Liang Tang, Liang Huang, Cong-Yang Zhou, Ji-Xiong Xu

**Affiliations:** ^1^Department of Internal Medicine, First Affiliated Hospital of Nanchang University, Nanchang, Jiangxi 330006, China; ^2^Department of Respiratory Medicine, First Affiliated Hospital of Nanchang University, Nanchang, Jiangxi 330006, China; ^3^Department of Endocrinology and Metabolism, Third Hospital of Nanchang, Nanchang, Jiangxi 330006, China; ^4^Department of Endocrinology and Metabolism, First Affiliated Hospital of Nanchang University, Nanchang, Jiangxi 330006, China

## Abstract

The S100 protein family is involved in cancer cell invasion and metastasis, but its prognostic value in non-small-cell lung cancer (NSCLC) has not been elucidated. In the present study we investigated the prognostic role of mRNA expression of each individual S100 in NSCLC patients through the Kaplan–Meier plotter (KM plotter) database. Expression of 14 members of the S100 family correlated with overall survival (OS) for all NSCLC patients; 18 members were associated with OS in adenocarcinoma, but none were associated with OS in squamous cell carcinoma. In particular, high mRNA expression level of S100B was associated with better OS in NSCLC patients. The prognostic value of S100 according to smoking status, pathological grades, clinical stages, and chemotherapeutic treatment of NSCLC was further assessed. Although the results should be further verified in clinical trials our findings provide new insights into the prognostic roles of S100 proteins in NSCLC and might promote development of S100-targeted inhibitors for the treatment of NSCLC.

## 1. Introduction

Lung cancer is the leading cause of cancer-related mortality worldwide and is generally classified as small-cell lung cancer and non-small-cell lung cancer (NSCLC), which is mainly composed of lung adenocarcinoma and lung squamous cell carcinoma. With early and aggressive treatment, the 5-year survival of lung cancer patients is greater than 50%, but once metastatic disease occurs the survival rate drops to 5% [1, 2]. Most patients with NSCLC die due to recurrent disease. Therefore, identification of potential prognostic markers is a matter of great clinical urgency for patients with NSCLC.

The S100 calcium binding protein family, including more than 25 known members the first of which was reported by Moore in 1965, is only expressed in vertebrates and plays a key role in modulating the transmission of various cellular signals [3]. Many studies suggest that the expression of S100 family members is altered in numerous human cancers [4–7] and recent studies have reported that S100 protein may be associated with tumor metastasis [8–10].

In NSCLC, several S100 family members (S100A2, S100A4, S100A7, S100A10, S100A11, and S100A16) have been shown to be related to poor prognosis in different studies [5, 11–13], although the role of the majority of S100 proteins in NSCLC has not been reported.

The Kaplan–Meier (KM) plotter database was generated using gene expression data and survival information downloaded from the Gene Expression Omnibus (GEO) (https://www.ncbi.nlm.nih.gov/geo/). The database contains overall survival (OS) data for 1,926 NSCLC patients [14]. To date, several potential cancer-associated genes have been reported using the KM plotter for breast cancer [15–17], ovarian cancer [18], and gastric cancer [19, 20] in addition to NSCLC. In this study, we assessed the prognostic role of mRNA expression of each individual member of the S100 family in NSCLC patients using the KM plotter database.

## 2. Materials and Methods

The correlation of individual S100 mRNA expression with OS was analyzed using an online database that was established using gene expression data and survival information of lung cancer patients downloaded from the GEO [14]. Currently, breast cancer [21], ovarian cancer [22], gastric cancer [23], and lung cancer [14] databases have been generated. The database contains a collection of clinical data including histology, stage, grade, gender, and smoking history, and treatment groups include surgery, chemotherapy, and radiotherapy. Briefly, 20 individual members of the S100 family were entered into the database (http://kmplot.com/analysis/index.php?p=service&cancer=lung) to obtain KM survival plots. The requested mRNA expression above or below the median classified the cases into a high expression group and low expression group. These cohorts were compared with a Kaplan–Meier survival plot, and hazard ratio (HR), 95% confidence interval (CI), and log rank *p* value were determined and displayed on the webpage. A *p* value < 0.01 was considered statistically significant to reduce the false-positive rate.

## 3. Results

### 3.1. Prognostic Value of S100 Members in All NSCLC Patients

**Figure 1:**
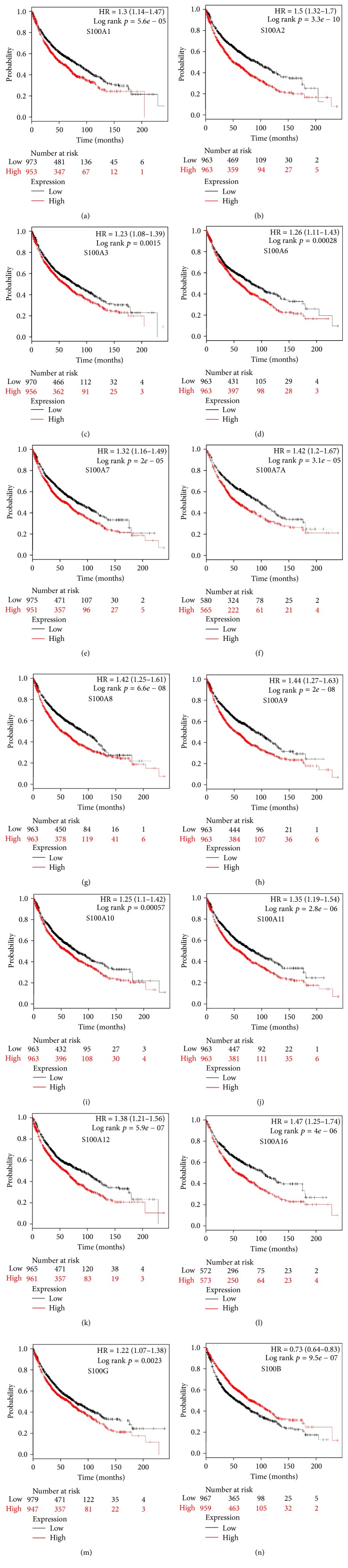
Prognostic value of S100 mRNA expression from the database. (a–n) Survival curves of S100A1 (Affymetrix IDs are valid: 205334_at), S100A2 (204268_at), S100A3 (206027_at), S100A6 (217728_at), S100A7 (205916_at), S100A7A (232170_at), S100A8 (202917_s_at), S100A9 (203535_at), S100A10 (200872_at), S100A11 (200660_at), S100A12 (205863_at), S100A16 (227998_at), S100G (207885_at), and S100B (209686_at) are plotted for all NSCLC patients (*n* = 1,926). Among them, only S100B mRNA expression was associated with better OS; the other S100 members were associated with worse OS.

**Figure 2:**
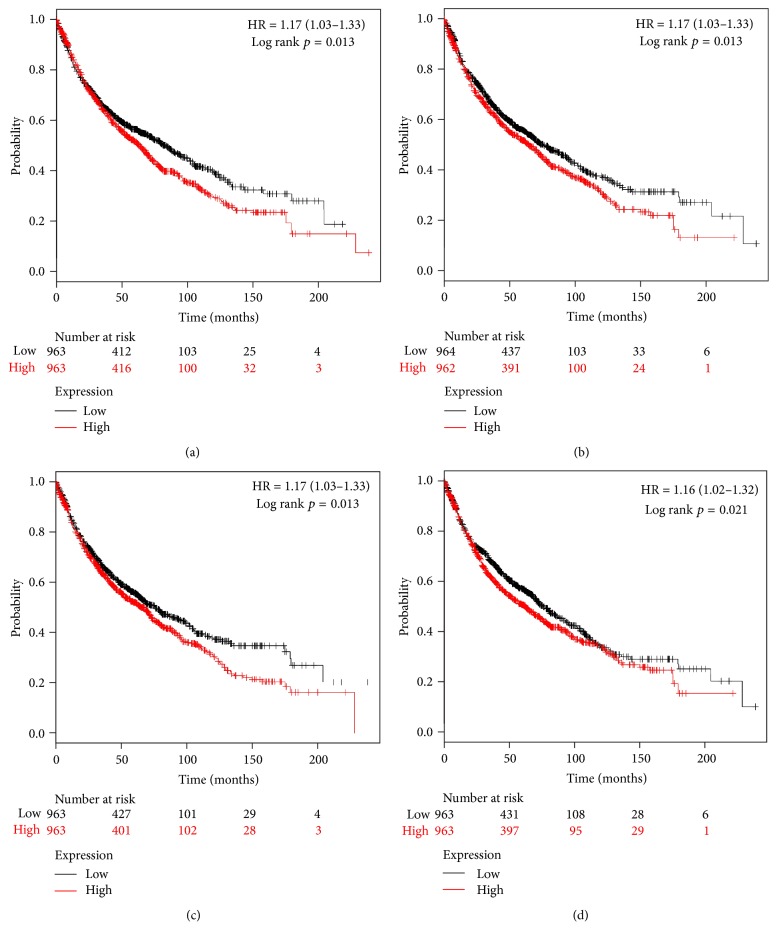
Survival curves for (a) S100A4 (Affymetrix ID is valid: 203186_s_at), (b) S100A13 (202598_at), (c) S10014 (218677_at), and (d) S100P (204351_at) plotted for all NSCLC patients (*n* = 1,926). All were modestly associated with worse OS.

Twenty S100 family members present in NSCLC patients were found in the database (http://kmplot.com), and we determined the prognostic value of mRNA expression of each one individually. Among these 20 S100 members, 14 were significantly associated with prognosis for all NSCLC patients (Figure 1). The survival curves (Figures 1(a)–1(n)) revealed that high mRNA expression of S100B was associated with better prognosis (Figure 1(n), HR = 0.73, 95% CI: 0.64–0.83, and *p* = 0.0000). The other 13 members were associated with worse prognosis (Figures 1(a)–1(m)). S100A4 (HR = 1.17, 95% CI: 1.03–1.33, and *p* = 0.0130), S100A13 (HR = 1.17, 95% CI: 1.03–1.33, and *p* = 0.0130), S100A14 (HR = 1.17, 95% CI: 1.03–1.33, and *p* = 0.0130), and S100P (HR = 1.16, 95% CI: 1.02–1.32, and *p* = 0.0210) were modestly associated with poor survival (Figures 2(a)–2(d)), whereas S100A5 (HR = 1.12, 95% CI: 0.98–1.27, and *p* = 0.0850) and S100Z (HR = 0.90, 95% CI: 0.76–1.06, and *p* = 0.2100) were not correlated with OS.

### 3.2. Prognostic Values of S100 Members in Different NSCLC Subtypes

The prognostic value of S100 family members was assessed in different intrinsic subtypes of NSCLC, including squamous cell carcinoma and adenocarcinoma. In squamous cell carcinoma, none of high mRNA expression levels of the S100 family members correlated with OS.

In adenocarcinoma patients, S100B mRNA expression level was associated with better OS. S100G (HR = 1.29, 95% CI: 1.03–1.63, and *p* = 0.0290) and S100Z (HR = 0.91, 95% CI: 0.71–1.16, and *p* = 0.4300) were not significantly related to prognosis in adenocarcinoma and expression of the other 17 S100 members correlated with worse OS.

### 3.3. Prognostic Values of S100 Members in NSCLC Patients according to Clinicopathological Features and Treatment

Next, we determined the correlation of S100 with the patients' smoking status, pathological grades, clinical stages, and different chemotherapeutic treatments. As shown in Table 1, high mRNA expression of S100A14 (HR = 1.31, 95% CI: 1.07–1.62, and *p* = 0.0098) and S100P (HR = 1.38, 95% CI: 1.12–1.7, and *p* = 0.0022) correlated with worse OS in patients with a history of smoking. High mRNA expression of S100A5 (HR = 2.73, 95% CI: 1.47–5.07, and *p* = 0.0004), S100A6 (HR = 3.53, 95% CI: 1.87–6.65, and *p* = 0.0000), S100A13 (HR = 3.32, 95% CI: 1.79–6.16, and *p* = 0.0001), S100A16 (HR = 5.12, 95% CI: 1.75–14.99, and *p* = 0.0009), and S100G (HR = 2.93, 95% CI: 1.62–5.31, and *p* = 0.0002) correlated with worse OS in patients without smoking history. High S100A2, S100A7, S100A8, S100A9, S100A11, and S100A12 mRNA expression was linked to worse OS in patients with and without smoking history. However, only S100B mRNA expression level was associated with better OS in patients with smoking history (HR = 0.71, 95% CI: 0.58–0.87, and *p* = 0.0012).

None of high mRNA expression levels of the S100 family members correlated with OS. The expression of S100P (HR = 1.4, 95% CI: 1.02–1.93, and *p* = 0.0340) and S100B (HR = 0.47, 95% CI: 0.24–0.93, and *p* = 0.025) was modestly associated with OS in patients with grade II and III lung cancer, respectively.

High mRNA expression of most of the S100 family members was associated with OS in clinical stage I patients except for S100A14, S100G, and S100Z. Only high RNA expression of S100B was linked to better prognosis (HR = 0.59, 95% CI: 0.45–0.78, and *p* = 0.0001). High mRNA expression of S100A1 (HR = 1.65, 95% CI: 1.14–2.38, and *p* = 0.0068), S100A6 (HR = 1.69, 95% CI: 1.16–2.45, and *p* = 0.0053), and S100B (HR = 0.58, 95% CI: 0.40–0.84, and *p* = 0.0032) was associated with OS in clinical stage II, and high S100B mRNA expression still correlated with better prognosis in this subgroup. However, none of the S100 mRNAs correlated with OS in clinical stage III patients.

Only S100A11 (HR = 1.89, 95% CI: 1.26–2.84, and *p* = 0.0018) significantly correlated with survival in patients treated with chemotherapy. High S100A12 (HR = 1.55, 95% CI: 1.11–2.17, and *p* = 0.0095) and S100G (HR = 1.72, 95% CI: 1.23–2.41, and *p* = 0.0013) mRNA expression were linked with worse OS in patients who did not undergo chemotherapy.

## 4. Discussion

In this study, we investigated the expression level of each individual S100 family member and its prognostic value in NSCLC. Among them, 14 members were significantly associated with prognosis, but only S100B was significantly associated with better prognosis. However, the molecular mechanisms by which S100 proteins contribute to disease aggression are not understood. Many studies have suggested that the expression of S100 protein in NSCLC is related to prognosis. High expression of S100A4 has been observed in NSCLC and was associated with differentiation and metastasis of tumor cells [11]; however, our results showed that S100A4 was not significantly related to OS in NSCLC.

Among the 14 S100 family members mentioned above, S100A1, S100A3, S100A7A, S100A12, and S100G display increased expression in several cancers [8, 24–26]. Our results suggested that high mRNA expression of these members was significantly associated with worse prognosis in all NSCLC patients. However, the roles of S100A1, S100A3, S100A7A, S100A12, S10016, and S100G proteins in lung cancer are rarely reported. In this study, we selectively discuss the other eight members.

High protein expression of S100A2 has been confirmed in the early stage of NSCLC [27] and is a prognostic marker associated with poor survival and a high risk of metastasis [4, 28]. Furthermore, S100A2 is considered a novel transcriptional target of p53 homologues, playing a pivotal role in regulating the cell cycle and triggering apoptotic programmed cell death in response to DNA damage or stress [29–31]. Epidermal growth factor receptor (EGFR) signaling is the main regulatory pathway for S100A2 transcription in human keratinocytes and other epithelial cells [32]. In addition, S100A2 induced epithelial-mesenchymal transition (EMT), increased invasive capability, loosened colony morphology in soft agar, and enhanced Akt phosphorylation in A549 lung cancer cells to promote tumorigenic actions and tumor growth [33]. Our data suggested that high mRNA expression of S100A2 was associated with worse OS in patients with stage I NSCLC.

The expression and prognostic role of S100A6 have been identified in thyroid [34], colorectal [35], and osteosarcoma [36] cancers. In NSCLC, S100A6 is associated with cell proliferation [37, 38]. Furthermore, high S100A6 protein level could lead to cell apoptosis by facilitating the apoptotic action of p53 [39]. Consistent with these findings, increased mRNA expression of S100A6 was significantly associated with poor prognosis in NSCLC patients in our study.

Expression of S100A7 mRNA and protein is increased mainly in squamous cell carcinoma tissues and breast cancer [40–42]. S100A7 was demonstrated to be involved in cancer growth and metastasis through modulation of the tumor microenvironment [43, 44]. Knockdown of S100A7 attenuated lung cancer growth by disruption of nuclear factor-*κ*B activity, and S100A7 was reported as a potential diagnostic marker in lung squamous cell carcinoma [45]. Overexpression of S100A7 was associated with poor survival in SCC cells [45]. In this study, high mRNA expression of S100A7 was associated with worse OS in adenocarcinoma, but not in squamous cell carcinoma.

S100A10 forms a heterotetramer with annexin A2, which acts as a receptor of plasminogen and is involved in the conversion of plasminogen to plasmin [46]. Plasmin contributes to degradation of the basement membrane and ECM [46, 47]. S100A10 was overexpressed in renal cell carcinoma, anaplastic thyroid carcinoma, gallbladder, and colorectal cancer [48–51]. Decreased expression of S100A10 in HT1080 fibrosarcoma cells and colorectal cancer cells weakened their invasiveness and metastatic potential, suggesting that S100A10 contributes to cancer cell invasiveness [52, 53]. Katono et al. reported that S100A10 was highly expressed in lung adenocarcinomas and suggested that S100A10 may enhance the invasiveness of tumor cells by increasing plasmin production [13]. We reported a similar result, with high S100A10 expression related to poorer prognosis in NSCLC patients.

Upregulation of S100A11 plays a major role in lung cancer progression [54]. S100A11 protein was selectively expressed in NSCLC and displayed a particularly prominent effect in KRAS-mutated lung adenocarcinomas [5, 54]. It was reported that the A549 and LTEP-a-2 cell lines, which have lost S100A11 expression, show a marked suppression in tumor growth, and S100A11 knockdown also significantly inhibited tumor growth in vivo [54]. Zagryazhskaya et al. reported that S100A11 might be involved in regulation of chemoresistance of NSCLC cells [55]. We showed that high mRNA expression of S100A11 was associated with worse OS in NSCLC patients treated with chemotherapy and those with early-stage disease.

The protein heterodimer of S100A8 and S100A9 has been implicated in tumor development and progression [56]. The S100A8/A9 heterodimer has been shown to promote accumulation of myeloid-derived suppressor cells (MDSCs) in the primary tumor and their recruitment to premetastatic lungs [57, 58]. MDSCs function to induce expansion and local accumulation of regulatory T cells and suppress the antitumor immune response of natural killer cells [58, 59]. Eisenblaetter et al. reported increased S100A8/A9 levels in premetastatic lung tissue and accumulation of MDSC-like monocytes [60]. The authors proposed S100A8/A9 as a potent imaging biomarker for tumor-mediated immune remodeling [60]. In the present study, high expression levels of S100A8 and S100A9 were negative prognostic markers in NSCLC patients.

S100B is mainly found in Schwann cells of the peripheral nervous system and extraneuronally in melanocytes, adipocytes, and chondrocytes [61, 62] and is implicated in regulating enzyme activities, cell growth, and differentiation [63, 64]. S100B plays a prognostic role in the majority of brain metastases of melanoma [65]. Similarly, elevated serum S100B levels in NSCLC might be associated with the development of brain metastasis [66, 67]. S100B overexpression could contribute to cancer progression by interfering with p53 activity [64, 66]. These results imply that S100B may be a predictor of poor prognosis in lung cancer. However, contrary to our expectation, high expression of S100B in lung cancer was associated with better OS in this study. There are two possible reasons for this discrepant result: first, we obtained this result using the retrospective Kaplan–Meier plotter database and second study of S100B has mainly focused on in vitro systems and a future clinical trial is needed to validate the findings.

Tobacco smoke contains many classes of carcinogens. Nicotine, one of the carcinogens, is the addictive and most active component of tobacco smoke. Although it is not directly involved in tumorigenesis, it has been shown to promote tumor growth and metastasis [68, 69]. In vivo, nicotine can be converted to cotinine, which has tumor-promoting effects in the lung such as inducing abnormal cell formation and proliferation, promoting tumor growth, and inhibiting apoptosis [68, 70]. Furthermore, nicotine genotoxicity was described through activation of cell surface nicotinic acetylcholine receptors (nAChRs), which led to increased levels of reactive oxygen species (ROS) [71, 72]. At present, there are no reports suggesting a direct correlation between nicotine and S100 proteins in NSCLC. Recently, nicotine was reported to promote NSCLC growth and metastasis by inducing the secretion of stem cell factor [73]. Cigarette smoke induces airway inflammation by downregulating S100A8/A9 [74]. In our study, expression of S100A2, S100A7, S100A8, S100A9, S100A11, and S100A12 but not that of S100A1, S100A3, S100A4, S100A7A, S100A10, and S100Z correlated with smoking status of NSCLC patients; S100A5, S100A6, S100A13, S100A16, and S100G were associated with worse survival in nonsmoking NSCLC patients; and only S100B, but S100A14 and S100P, was associated with better survival in smoking NSCLC patients.

## 5. Conclusions

In the present study, the prognostic value of mRNA expression of 20 members of the S100 family in NSCLC patients was assessed using the KM plotter database. Among them, 14 members were associated with prognosis in all NSCLC patients. However, only increased S100B mRNA expression was significantly associated with better OS in all NSCLC patients. The prognostic value of the S100 protein should be further evaluated in clinical studies. These results will be helpful for investigating the relationship between proteins and disease and their roles in different signaling pathways. Our study provides new insights into the prognostic functions of S100 proteins in NSCLC and might promote development of S100 targeted inhibitors for the treatment of NSCLC.

## Figures and Tables

**Table 1:** Correlation of S100 member expression with smoking status of NSCLC patients. Expression of S100A2, S100A7, S100A8, S100A9, S100A11, and S100A12 correlated with smoking status of NSCLC patients; S100A5, S100A6, S100A13, S100A16, and S100G were associated worse survival in nonsmoking NSCLC patients; and only S100B, but S100A14 and S100P, was associated with better survival in smoking NSCLC patients.

S100 family	Affymetrix IDs	Smoking status	HR	95% CI	*p* value
S100A1	205334_at	Never smoked	2.1	(1.17, 3.77)	0.0110
		Smoked	1.21	(0.98, 1.48)	0.0750
S100A2	204268_at	Never smoked	4.1	(2.1, 8)	0.0000
		Smoked	1.47	(1.19, 1.81)	0.0003
S100A3	206027_at	Never smoked	1.91	(1.07, 3.38)	0.0250
		Smoked	1.15	(0.93, 1.41)	0.2000
S100A4	203186_s_at	Never smoked	2.06	(1.14, 3.7)	0.0140
		Smoked	1.23	(1, 1.51)	0.0500
S100A5	207763_at	Never smoked	2.73	(1.47, 5.07)	0.0009
		Smoked	1.13	(0.92, 1.39)	0.2400
S100A6	217728_at	Never smoked	3.53	(1.87, 6.65)	0.0000
		Smoked	1.29	(1.05, 1.59)	0.0160
S100A7	205916_at	Never smoked	4.37	(2.35, 8.11)	0.0000
		Smoked	1.34	(1.09, 1.65)	0.0053
S100A7A	232170_at	Never smoked	1.12	(0.5, 2.5)	0.7800
		Smoked	1.55	(1.03, 2.34)	0.0330
S100A8	202917_s_at	Never smoked	2.86	(1.54, 5.3)	0.0005
		Smoked	1.76	(1.42, 2.17)	0.0000
S100A9	203535_at	Never smoked	3.18	(1.71, 5.9)	0.0001
		Smoked	1.55	(1.26, 1.91)	0.0000
S100A10	200872_at	Never smoked	1.66	(0.93, 2.94)	0.0810
		Smoked	1.22	(0.99, 1.5)	0.0620
S100A11	200660_at	Never smoked	4	(2.09, 7.67)	0.0000
		Smoked	1.36	(1.11, 1.68)	0.0033
S100A12	205863_at	Never smoked	2.76	(1.5, 5.05)	0.0006
		Smoked	1.37	(1.11, 1.69)	0.0028
S100A13	202598_at	Never smoked	3.32	(1.79, 6.16)	0.0001
		Smoked	1.16	(0.94, 1.43)	0.1600
S100A14	218677_at	Never smoked	1.8	(1.02, 3.2)	0.0410
		Smoked	1.31	(1.07, 1.62)	0.0098
S100A16	227998_at	Never smoked	5.12	(1.75, 14.99)	0.0009
		Smoked	1.29	(0.85, 1.95)	0.2300
S100B	209686_at	Never smoked	0.77	(0.44, 1.35)	0.3700
		Smoked	0.71	(0.58, 0.87)	0.0012
S100G	207885_at	Never smoked	2.93	(1.62, 5.31)	0.0002
		Smoked	1.25	(1.02, 1.54)	0.0340
S100P	204351_at	Never smoked	1.22	(0.7, 2.15)	0.4800
		Smoked	1.38	(1.12, 1.7)	0.0022
S100Z	1554876_a_at	Never smoked	0.82	(0.37, 1.83)	0.6300
		Smoked	1.21	(0.81, 1.82)	0.3500
